# Expansion of *Drosophila* haemocytes using a conditional *GeneSwitch* driver affects larval haemocyte function, but does not modulate adult lifespan or survival after severe infection

**DOI:** 10.1242/jeb.249649

**Published:** 2025-05-06

**Authors:** Dan J. Hayman, Lola M. Morrin, Sudipta Halder, Eleanor J. Phillips, Mirre J. P. Simons, Iwan R. Evans

**Affiliations:** ^1^ School of Biosciences, University of Sheffield, Sheffield S10 2TN, UK; ^2^ Division of Clinical Medicine, School of Medicine and Population Health and Bateson Centre for Disease Mechanisms, University of Sheffield, Sheffield S10 2TN, UK

**Keywords:** *Drosophila*, Macrophage, Haemocyte, Ageing, Immunity

## Abstract

Macrophages are responsible for diverse and fundamental functions in vertebrates. *Drosophila* blood cells (haemocytes) are dominated by cells bearing a striking homology to vertebrate macrophages (plasmatocytes). The importance of haemocytes has been demonstrated previously, with immune and developmental phenotypes observed upon haemocyte ablation. Here, we show that we can increase *Hemolectin* (*Hml*)-positive cell numbers using a constitutively active form of *ras* and ablate *Hml*-positive cell numbers using the pro-apoptotic transgene *bax*. However, compared with larvae, total blood cell numbers in adults were not significantly affected by experimental expansion or ablation, implying the existence of feedback mechanisms regulating haemocyte numbers. No effect on lifespan was observed from driving *ras* and *bax* in *Hml*-positive cells via a conditional approach (*Hml*-*GeneSwitch*). Using constitutive expression, we observed differences in lifespan; however, we attribute this to differences in genetic background. Additionally, no effect of either transgene was observed upon infection with a high dose of two different bacterial species, although pupal lethality was observed upon expansion of *Hml*-positive cells in a self-encapsulation mutant genetic background. The latter confirms that changes in *Hml*-positive cell numbers can result in phenotypes. The lack of adult phenotypes could be due to the strength of experimental manipulations or compensation via feedback mechanisms operating to regulate total blood cell numbers. Our study demonstrates the importance of conditional approaches to modulate haemocyte cell numbers, allowing for more precise study of innate immune function. This strategy could be especially fruitful to uncover mechanisms regulating total blood cell numbers across development and ageing.

## Teaser

**Summary:** Increasing the number of fly macrophages using a conditional expression strategy alters larval immune phenotypes and reveals feedback mechanisms regulating blood cell numbers *in vivo*.

## INTRODUCTION

Vertebrate macrophages enact diverse functions, including clearance of infections, removal of apoptotic corpses and tissue remodelling as part of development (Wynn et al., 2013). Thus, macrophages are by necessity highly heterogeneous populations of cells, showing high levels of variation at both functional and transcriptomic levels (Murray et al., 2014; Colin et al., 2014; Gordon and Taylor, 2005; Gordon et al., 2014). As a result of this heterogeneity, the study of macrophages in vertebrate systems is challenging, especially considering the necessity to also consider contributions from the adaptive immune system. Therefore, the use of invertebrate models which represent vertebrate macrophages well is of great value to the field of immunology. The fruit fly *Drosophila melanogaster* is such a model system. Flies lack an adaptive immune system and solely harbour an innate immune system, which consists of circulatory blood cells known as haemocytes (Buchon et al., 2014; Holz et al., 2003). Broadly, haemocytes consist of three distinct cell types: crystal cells, lamellocytes and plasmatocytes. The *Hml* promoter is active in intermediate prohaemocytes, which are precursor cells within the cortical zone of the lymph gland (Sinenko, 2024), as well as most plasmatocytes and crystal cells, but not lamellocytes (Goto et al., 2003). This last group of cells is responsible for the encapsulation of large foreign objects. Functionally, crystal cells are thought to mediate melanisation, whereas plasmatocytes, which dominate the haemocyte population in quantity, are considered highly homologous in function to vertebrate macrophages (Yoon et al., 2023; Buchon et al., 2014; Wood and Jacinto, 2007; Evans et al., 2003). These cells are able to migrate to wound sites, engulf pathogens and deposit extracellular matrix, all of which are essential characteristics of macrophages, as well as displaying transcriptomic and functional heterogeneity (Coates et al., 2021; Ratheesh et al., 2015). Considering these features of this class of *Drosophila* haemocyte, the fruit fly has been utilised frequently as a model of macrophages and their associated behaviours (Ratheesh et al., 2015; Wood and Jacinto, 2007).

Considering the wide array of roles for which *Drosophila* haemocytes are responsible, it would seem logical for flies genetically engineered to lack haemocytes to be non-viable. Interestingly however, the developmental lethality of flies in which haemocytes are ablated appears to vary depending on the specific genetic driver used. Different studies have used different parts of the promoter region of the *Hemolectin* (*Hml*) gene to genetically ablate haemocytes. Flies in which haemocytes are ablated using the *HmlΔ* driver complete development, albeit with an approximately halved ratio of successful eclosion from pupae relative to control flies, but this phenotype is rescued by germ-free or antibiotic-supplemented conditions (Shia et al., 2009; Charroux and Royet, 2009; Arefin et al., 2015; Defaye et al., 2009; Ayyaz et al., 2015). In some studies, melanotic spots were observed in flies in which haemocytes had been ablated, proposed to be the result of a lamellocyte expansion in *HmlΔ*-positive cell-ablated conditions (Defaye et al., 2009; Arefin et al., 2015). Comparatively, flies in which haemocytes are ablated using a different region of the *Hml* promoter, named *Hml*^P2A^, are severely pupal lethal, and are unable to be rescued (Stephenson et al., 2022). However, haemocyte ablation has not been investigated intensively with condition-dependent drivers. The benefit of conditional drivers is that all genetics are controlled for, with a genetic system conditionally activated by specific drugs (Clausen et al., 1999; Bockamp et al., 2008; Osterwalder et al., 2001) or environmental variables, such as temperature (McGuire et al., 2003; del Valle Rodríguez et al., 2011) and light (de Mena and Rincon-Limas, 2020). A small number of experiments have used a temperature-sensitive Gal80 to ablate haemocytes conditionally (Defaye et al., 2009; Monticelli et al., 2024; Ayyaz et al., 2015), one of which revealed a reduced post-infection survival phenotype upon conditional haemocyte ablation (Defaye et al., 2009). Another showed that ablation of haemocytes impairs intestinal regeneration following oxidative stress-mediated damage or infection with the pathogens *Erwinia carotovora carotovora* strain 15 or *Pseudomonas entomophila* (Ayyaz et al., 2015). However, never before has a non-temperature-dependent conditional approach been used to ablate haemocytes. In addition, it has not been established whether haemocyte function is altered by increasing the number of cells; in other words, the literature suggests that a lack of haemocytes negatively affects haemocyte-driven functions, but does an excess of haemocytes produce the inverse of this?

Here, we show the importance of the use of conditional drivers, especially for lifespan as a phenotype. Lifespan is highly polygenic making it critical to homogenise genetic backgrounds or use conditional systems where possible (Ford and Tower, 2005). Our results differed between experiments using constitutive and conditional drivers, despite both systems using the same promoter region, *HmlΔ*. The constitutive *HmlΔ* driver (*HmlΔ*-*Gal4*) successfully expanded and ablated *Drosophila Hml-*positive haemocytes through UAS-regulated *ras85D^V12^* and *bax* transgenes, respectively, and this correlated with an apparent modulation of lifespan. However, with our newly generated conditional driver, *HmlΔ*-*GeneSwitch*, no lifespan phenotype was observed, in spite of a modulation of *Hml*-positive cell numbers comparable to the constitutive driver experiments. Surprisingly, altering *Hml*-positive cell numbers conditionally did not change post-infection survival, raising the possibility that somehow the conditional modulation of *Hml*-positive haemocyte numbers did not change overall function. However, expansion of the *Hml*-positive cell population did modulate haemocyte function in larvae, which we demonstrated using a genetic model of self-encapsulation (Mortimer et al., 2021).

Bleeding larvae to obtain haemocyte populations revealed a large increase in overall haemocyte cell numbers when *HmlΔ*-*GeneSwitch* was used to drive *ras85D^V12^*. However, in adult bleeds this was not the case, even though the *Hml*-positive population did grow in number *in vivo*. Conversely, our ablation conditions in adults did result in low numbers of *Hml*-positive cell numbers, but no change in overall bled cell numbers was observed. This suggests mechanisms that regulate total blood cell numbers could compensate for the ablation of *Hml*-positive haemocytes, despite strong initial manipulations in the larva. Our findings show that haemocyte numbers in larvae have functional relevance; more careful study of these functions *in vivo* will be possible using well-controlled conditional genetic systems such as presented herein.

## MATERIALS AND METHODS

### Fly culture conditions

All experimental flies were kept at 25°C. Flies were kept for mating for 2 days following eclosion and were subsequently sorted into sexes and the desired genotypes for the experiment. Fly food consisted of the following components, as previously described (McCracken et al., 2020a,b): 8% yeast (Kerry), 13% table sugar (Tate and Lyle), 6% cornmeal (Triple Lion Medium Cornmeal), 1% agar (United States Biological) and 0.225% nipagin (Sigma-Aldrich) (all w/v). In the case of growing bottles, 0.4% (v/v) propanoic acid (Sigma-Aldrich) was also added. Where the GeneSwitch construct was utilised, food was supplemented with RU486 (Fisher; 200 μmol l^−1^ final media concentration; dissolved in 8.6 ml absolute ethanol (Fisher) per 1 litre of fly media and mixed into the media) or control food lacking RU486 (but still containing 8.6 ml absolute ethanol per 1 litre of fly media). The food used for control and RU486 food was split from a single media preparation, meaning the exact same food was used for both conditions at the same time. For all experiments using *HmlΔ-GeneSwitch*, RU486 was supplied to flies throughout development.

### *Drosophila* genotypes utilised

All fly genotypes utilised in this study are listed in Table 1, including source, where applicable. *HmlΔ-GeneSwitch* transgenic flies available on request.

**Table 1.** *Drosophila melanogaster* lines used in this research, specifying their use and source

Genotype	Purpose	Source/Bloomington stock no.
*w^1118^*	To enable selection on eye colour and normalise the X chromosome background as much as possible between lines used.	BL3605
*yw*	Used as a genetic control for constitutive crosses (Wessells et al., 2004; Phillips and Simons, 2024).	Wessells et al. (2004)
*w^1118^;;UAS-Stinger*	Males used as controls in the cross with the *HmlΔ-Gal4* and *HmlΔ-GeneSwitch* driver lines. Also crossed into *UAS-ras85D^V12^* and *UAS-bax* lines.	BL84278 (Barolo et al., 2000)
*w^1118^;UAS-ras85D^V12^;UAS-Stinger*	Males crossed with the *HmlΔ* driver line to expand the haemocyte population.	BL64196 (Simcox et al., 2008)
*w^1118^;UAS-bax;UAS-Stinger*	Males crossed with the *HmlΔ* driver line to ablate the haemocyte population.	Gaumer et al. (2000)
*w^1118^;;HmlΔ-Gal4*	Females used to cross with *UAS-ras85D^V12^, UAS-bax* or control males to expand or ablate haemocytes in a constitutive manner.	BL30142 (Sinenko and Mathey-Prevot, 2004)
*w^1118^;;HmlΔ-GeneSwitch*	Females used to cross with *UAS-ras85D^V12^*, *UAS-bax* or control males to expand or ablate haemocytes in a conditional manner, regulated by the presence of RU486.	Generated in this study
*w^tuSz1^*	Temperature-sensitive mutant line which has a recessive self-encapsulation phenotype.	BL5834 (Suzuki, 1970)
*w;sna^Sco^/CyO dfd-GMR-nvYFP*	*CyO dfd-GMR-nvYFP* is a balancer chromosome variant of *CyO* which contains a selectable fluorescent eye marker. Used for crosses and balancing. *CyO dfd* throughout this body of work refers to *CyO dfd-GMR-nvYFP*.	BL23230 (Le et al., 2006)

### Generation of *HmlΔ*-*GeneSwitch* flies

The *ELAV-GeneSwitch* plasmid (Osterwalder et al., 2001), deposited by Haig Keshishian (Addgene plasmid #83957; RRID:Addgene_83957), was digested at 37°C for 1 h with *Bgl*II and *Psp*XI (New England BioLabs), following the manufacturer's standard protocol. Genomic DNA was isolated from 20 *Drosophila melanogaster w^1118^* strain flies, utilising the DNeasy Blood & Tissue Kit (QIAGEN), following the manufacturer's standard protocol. The *HmlΔ* promoter was subsequently amplified from 80 ng genomic DNA using Platinum SuperFi II Polymerase (Invitrogen), with the following primers (*Bgl*II and *Psp*XI restriction sites shown in lower case; additional bases added to maintain the GeneSwitch frame underlined), as previously used in the literature (Makhijani et al., 2011): F primer 5′-CGGTCACTcctcgaggCAAAAGTTATTTCTGTAGGC-3′; R primer 5′-CGGTAACTagatctGGCTGCTGGGAGTCCATTTTGTTAGGCTAATCGGAAATTG-3′. The amplified *HmlΔ* promoter and digested *ELAV-GeneSwitch* plasmid were each run on a 1% (w/v) agarose TAE gel at 80 V for 1 h and gel purified using the QIAquick Gel Extraction Kit (QIAGEN). Ligation of the insert and backbone was undertaken at 4°C for 16 h followed by room temperature (22°C) for 2 h, at a molar ratio of 10:1 (insert:backbone, using 50 ng insert DNA), performed using 1 U T4 DNA ligase (Invitrogen), in a 20 µl reaction. TOP10 Competent Cells (ThermoFisher Scientific) were transformed with the ligation mix using the heat-shock method, recovered in a 37°C shaking incubator at 225 rpm for 1 h, and plated on agar plates supplemented with carbenicillin (100 µg ml^−1^), which were incubated at 37°C overnight. Discrete colonies were picked and following growth overnight in carbenicillin-supplemented LB broth at 37°C and 225 rpm, plasmid DNA was isolated using the QIAquick Spin Miniprep Kit (QIAGEN). The entire plasmid was sequenced using Oxford Nanopore long reads (Plasmidsaurus) and transformed into *w^1118^ Drosophila melanogaster* flies using P-element transposition (GenetiVision). Standard fly husbandry methodology was used to generate homozygous flies containing the *HmlΔ*-*GeneSwitch* construct, which were subsequently tested for their ability to drive the *UAS-Stinger* transgene in a haemocyte-specific manner. Plasmids used to generate transgenic flies available on request.

### Imaging and quantification of haemocytes *in vivo*

Prior to imaging, adult flies were immobilised by incubation at −20°C for 10 min. An MZ205 FA fluorescent dissection microscope with a 1× PLANAPO objective lens (Leica) was subsequently used to image individual flies on the GFP channel (ET-GFP filter), using LasX image acquisition software (Leica). The following settings were used: exposure=800 ms, gain=1.0, zoom=30×. Haemocyte numbers were quantified using the Find Maxima tool in Fiji (Schindelin et al., 2012), with Prominence set to 20. Differences between conditions were assessed using a two-way ANOVA. Tukey HSD *post hoc* tests were used to specifically assess the statistical significance of differences relative to control flies. Flies containing the *HmlΔ-Gal4* driver were imaged 1 day post-eclosion, whereas *HmlΔ-GeneSwitch* flies were imaged at 1 day post-eclosion to confirm the same effects were present as found using the *HmlΔ-Gal4* driver, but were also imaged in a separate experiment at 6 days post-eclosion. This latter time point both allowed flies to feed on RU486-supplemented food after pupation and allowed assessment of whether modulation of haemocyte numbers lasted for a prolonged period into adulthood. Of note here is that, with the *HmlΔ-Gal4* driver, cell numbers were not quantified further into adulthood, since this driver is too weak to accurately visualise Stinger with the imaging methods used.

To image haemocytes in larvae, wandering L3 larvae of the appropriate genotypes were selected and washed in distilled water and blotted dry. They were then imaged in distilled water on ice using the same microscope as above (2× PLANAPO objective lens, ET-GFP filter, exposure=300 ms, zoom=1.34, gain=3.0).

### Assessment of post-infection survival

*Pseudomonas entomophila* (a gift from Julia Cordero, University of Glasgow, UK) and *Staphylococcus aureus* (a gift from Pedro Vale, University of Edinburgh, UK) bacteria (Vodovar et al., 2005; Perochon et al., 2021) were individually grown overnight at 29°C in LB broth, from stocks frozen at −80°C, to an OD_600nm_ of 0.5. Flies were individually immobilised using a CO_2_ pad and a 0.15 mm diameter stainless steel insect minutien pin was washed in the bacterial stock, before being intrathoracically inserted into the immobilised fly for 2 s. Each fly was subsequently housed alone in a vial containing standard fly media [containing 8% (w/v) yeast] food, labelled with the time of infection and incubated in a temperature and humidity controlled room. The following day, flies were checked for death every 30 min from 1 h after the light turned on. Cox proportional hazard models were used to test the effect of RU486 in the context of each genotype. The ‘coxph’ function in the *survival* package in R (https://CRAN.R-project.org/package=survival) was used to assess statistical significance.

### Longevity experiments

Flies were transferred to a custom cage to minimise disturbances, as previously described (McCracken et al., 2020a; Good and Tatar, 2001). No more than 100 flies were housed in each cage, with sexes separated. Following caging, every other day, the number of deaths were recorded (and dead flies subsequently removed) and a fresh vial of food was provided, until no flies remained. In order to conditionally drive the *HmlΔ-GeneSwitch* construct throughout development, RU486-containing or control food was utilised from early larval stages. Proportional Cox hazard models were used between conditions in each genotype and each sex where applicable, including the ‘cage’ as a random effect to correct for pseudoreplication within cages (McCracken et al., 2020b; Therneau et al., 2003; Ripatti and Palmgren, 2000). The *coxme* package in R (https://CRAN.R-project.org/package=coxme) was used to assess statistical significance to correct for the shared environment of cage. This was used in place of the ‘coxph’ function as it allows for inclusion of between-cage effects, which are not relevant for post-infection assessment of survival where only one fly is kept in each vial. Flies that escaped or that were squashed were censored; no other exclusion criteria were applied here or elsewhere so long as animals were the correct genotype as per discrimination via balancer chromosomes and markers. Flies were randomly assigned to different population cages for ageing with different drug regimens.

### Assessment of survival and melanisation in the context of the *tuSz^1^* mutation

To visualise an active cell-based immunity response, we used the *tuSz^1^* mutation, which results in haemocytes incorrectly recognising fat body cells as non-self and leading to self-encapsulation and associated melanisation (Mortimer et al., 2021). Virgin females containing the *tuSz^1^* mutation (*w^tuSz1^*) were crossed with *w^1118^;If/UAS-ras85D^V12^;HmlΔ-GeneSwitch* males at 25°C on food containing either RU486 or control food. After 24 h at 25°C, parents were removed and the vial containing eggs switched to either 29°C to enable the temperature-sensitive *tuSz^1^* melanotic phenotype to manifest, or kept at 25°C as negative controls. To assess if expansion of haemocytes caused lethality, offspring on both diets at both temperatures were sexed and counted, as well as being genotyped using the visible marker *If* to discriminate the presence of *UAS-ras85D^V12^*. At the L3 stage of development, 27 male larvae on a control diet and 25 male larvae on an RU486-containing diet at 29°C were randomly picked and their melanisation level scored from 1 to 5, based on a novel, semi-quantitative scoring system (shown in Fig. 6; flies were scored blinded). Each larva was individually housed in a fresh vial and eventually checked for genotype upon eclosion. Flies which did not eclose were checked for their genotype, via *If*, by removing the puparium. Larvae were imaged on a MZ205 FA fluorescent dissection microscope with a 1× PLANAPO objective lens (Leica) on the brightfield channel, using LasX image acquisition software (Leica), with the following settings: exposure=700 ms, gain=1.0 and zoom=40×. To image in detail the fat body of developmentally arrested flies following puparium removal, the same microscope was utilised, but with zoom set to 120×.

### Dissection, staining, imaging and analysis of larval and adult *ex vivo* bleeds

Wandering L3 larvae of the appropriate genotype were selected and washed in distilled water, blotted dry and then transferred to 75 μm of S2 media [Schneiders medium (Merck) supplemented with 1× Pen/Strep (Gibco) and 10% heat-inactivated FBS (Gibco)] on ice in a Petri dish. Larvae were opened using size 5 forceps from their posterior spiracles towards the anterior end to release haemocytes into S2 media and then agitated for 10 s to release more tightly adhered cells. S2 media (75 μl) containing haemocytes was then transferred to a 96-well plate (Greiner) and a further 75 μl of S2 media added. For adult bleeds, flies were placed at −20°C for 3 min ahead of dissection to stop movement. Two newly-eclosed adult flies (day 1) of the same sex were then dissected in a single 75 μl droplet of S2 media on ice. Flies were decapitated and then cut longitudinally along their head, thorax and abdomen within that droplet and then an additional 75 µl S2 media was added. Carcasses were agitated ten times by pipetting before cell suspensions were transferred to a well in a 96-well plate. For both larval and adult bleeds, cells were allowed to adhere at room temperature and in the dark for 45–60 min before fixation.

Cells adhered to wells were fixed in 4% formaldehyde (diluted from 16% EM grade, Thermo Fisher) solution in PBS for 15 min, before permeabilisation using 0.1% Triton X-100 (Sigma) in PBS (Oxoid) for 5 min. Cells were stained using either TRITC–phalloidin (1:1000, Invitrogen; all dissections with the exception of *HmlΔ-Gal4*, constitutive L3 larvae) or Texas Red–Phalloidin (1:400, Invitrogen; *HmlΔ-Gal4*, constitutive L3 larvae) along with nucBlue nuclear stain (2 drops per ml, Invitrogen). PBS washes were performed after each step and cells were imaged in a final volume of 200 μl PBS using an MDX ImageExpress Hi-Content microscope (10× lens). DAPI, Cy3, Texas Red and GFP filter sets were used to image nucBlue, TRITC-phalloidin, Texas Red-phalloidin and Stinger fluorescence, respectively. Nine sites were imaged per well.

Images from at least six fields of view (FOV) per well were exported as Tiff files using ImageExpress software. For larval bleeds, cell counts per FOV were made from the DAPI/nucBlue channel images. These images were segmented (thresholded, fill holes, watershed) to create binary images, which were then counted automatically using the ‘analyse particles’ tool in Fiji. All contrast adjustment and thresholding were applied equally to each Tiff file within each coherent data set.

For adult dissections, contrast adjustments were applied to Tiff images for each channel to create an RGB merged image; as previously each channel was treated equally across experimental groups. Using the point selection tool and channels tool in Fiji the number of *Hml*-positive cells with nucBlue-stained nuclei were counted in the green/EGFP and DAPI channels; *HmlΔ-Gal4* or *HmlΔ-GeneSwitch* were used to drive expression from *UAS-Stinger* in these samples. Non *Hml*-positive haemocytes were then scored on the basis of cell morphology (F-actin staining via Phalloidin/red channel fluorescence) and the presence of nucBlue staining. Cells without nucBlue staining were excluded.

Statistical significance was calculated using ANOVAs for the conditional driver, with pairwise comparisons assessed using Tukey's HSD *post hoc* testing, or Student's two-tailed *t*-tests for constitutive data. Normality of the residuals from the ANOVA models was investigated visually using histograms as well as heteroscedasticity by using plotting of predicted values against residuals.

## RESULTS

### Constitutive and conditional *HmlΔ* drivers can expand or ablate *Hml*-labelled haemocyte populations

**Fig. 1.**
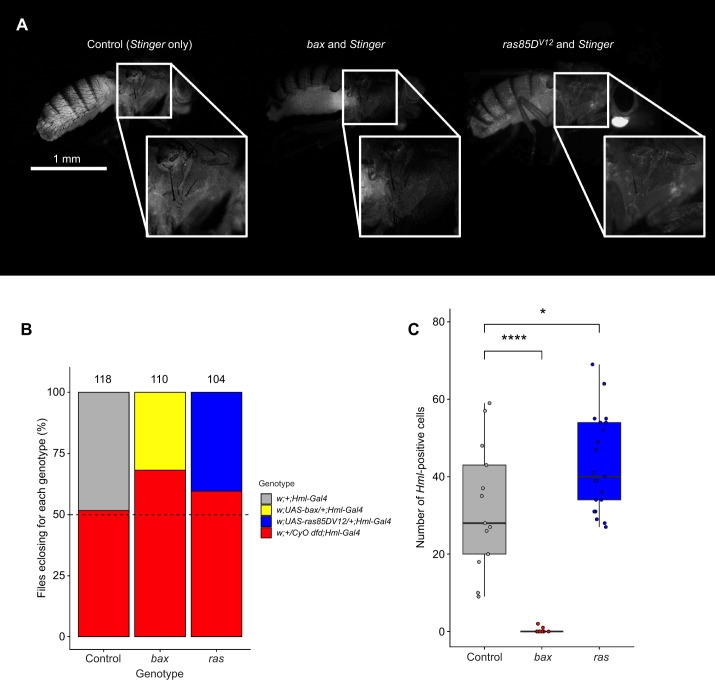
***HmlΔ*-*Gal4* can be used to expand or reduce numbers of *Hml*-labelled cells.** (A) Representative images of flies where Stinger only (Control), *bax* plus Stinger and *ras85D^V12^* plus Stinger were driven by the haemocyte-specific driver *HmlΔ-Gal4*. Driving *bax* or *ras85D^V12^* via *HmlΔ-Gal4* both produced viable progeny and modulated numbers of *HmlΔ*-labelled cells in female flies; *UAS*-*bax* reduced the labelled cell number to almost no cells, whereas *UAS-ras85D^V12^* increased the number of cells when imaged at 1-day post-eclosion. Cells labelled via expression from *UAS-Stinger*; Stinger is a nuclear-localised GFP, which enables visualisation of *HmlΔ*-positive cells. (B) Female *w^1118^;;HmlΔ-Gal4* flies were crossed with male flies of each of the following genotypes: *w^1118^;+/CyO dfd* (‘Control’), *w^1118^;UAS-bax/CyO dfd* (‘*bax*’) and *w^1118^;UAS-ras85D^V12^/CyO dfd* (‘*ras*’). The number of flies eclosing with *CyO dfd* was compared with those eclosing without it, so as to genotype the eclosing flies. The estimated ratio for each genotype was 50%, indicated by the dashed line, and each genotype was compared with the ratios observed in ‘Control’ flies using a χ^2^ test. The total number of progeny counted from each cross is shown above each bar. (C) Raw cell numbers detected via the Find Maxima tool from superficial, lateral images of adult flies at 1-day post-eclosion for each transgene are shown; *n*≥9 flies per condition; a one-way ANOVA with Tukey HSD *post hoc* tests was used to calculate statistical significance, for which *P≤0.05 and ****P≤0.0001. The exact genotypes used in A and C were as follows: ‘Control’=*w^1118^;;UAS-Stinger/HmlΔ-Gal4*; ‘*ras*’=*w^1118^;+/UAS-ras85D^V12^;UAS-Stinger/HmlΔ-Gal4*; ‘*bax*’=*w^1118^;+/UAS-bax;UAS-Stinger/HmlΔ-Gal4*. To ensure consistent egg density, 15 virgin female flies were used in each cross. In C, and all subsequent box plots in the main body figures, boxes represent the 25–75% range, lines represent the median, and whiskers represent the minimum–maximum range, excluding outliers; outliers were not excluded from statistical analyses.

We used *HmlΔ*-*Gal4* to constitutively drive either the pro-apoptotic gene *bax*, a potent activator of apoptotic pathways in the fly (Gaumer et al., 2000), in order to ablate *HmlΔ*-positive cells, or *ras85D^V12^*, a constitutively active mutant variant of *ras* used to drive expansion of the *HmlΔ*-positive cell population (Hobbs et al., 2016; Ashburner et al., 1999; Simcox et al., 2008). *Hml* was used to drive these *in vivo* manipulations as it is expressed in the majority of differentiated *Drosophila* haemocytes as well as in prohaemocyte precursor cells (Goto et al., 2003). Expression from *UAS-Stinger*, which encodes a nuclear-localised GFP (Barolo et al., 2000) enabled labelling of *Hml*-positive cells. Virtually no *Hml*-positive cells were visible in flies where *HmlΔ-Gal4* was used to drive *UAS-bax*, as found previously (Defaye et al., 2009), and increased numbers of *Hml*-positive cells were observed in flies where *HmlΔ*-*Gal4* was used to drive *UAS-ras85D^V12^* (Fig. 1A,C). We observed a slight increase in the developmental lethality of flies carrying *HmlΔ-Gal4* and either *UAS-bax* or *UAS-ras85D^V12^*, relative to control flies only carrying *HmlΔ-Gal4* (Fig. 1B). The increase in mortality in flies carrying *UAS*-*bax* flies achieved statistical significance (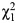
statistic=6.43, *P*-value=0.011), whereas the apparent increase in *UAS-ras85D^V12^* flies was not significant (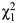 statistic=1.40, *P*-value=0.236).

**Fig. 2.**
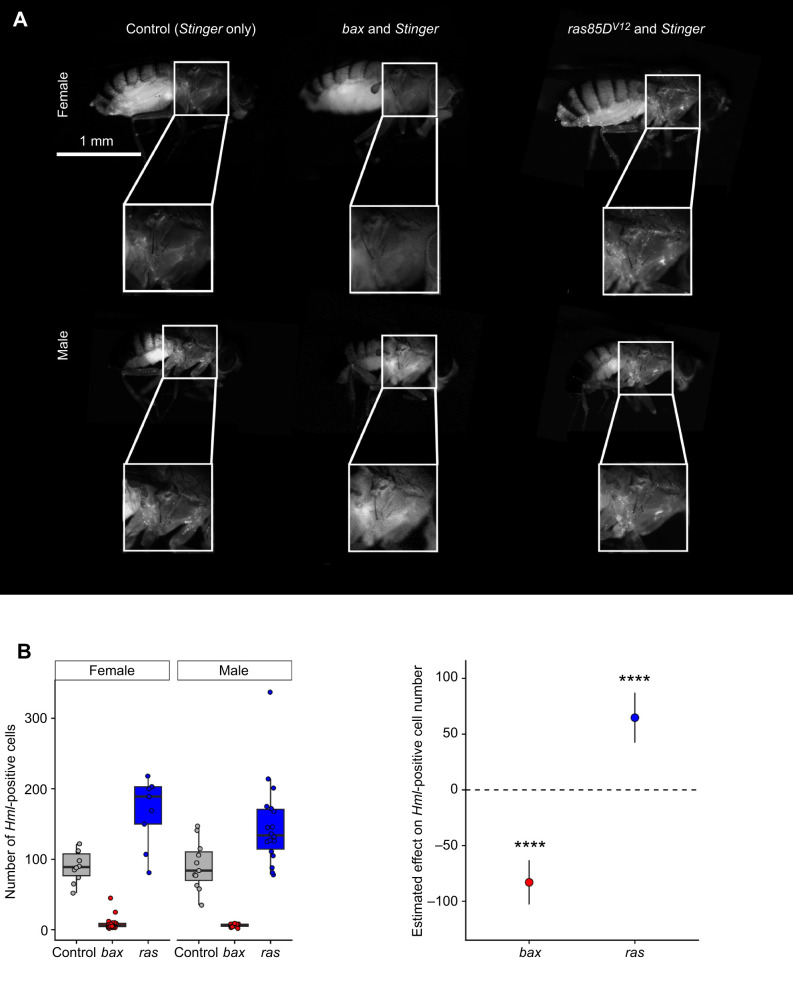
**The *HmlΔ*-*GeneSwitch* construct can be induced by RU486 and is able to modulate numbers of *Hml*-labelled cells.** (A) Driving *bax* or *ras85D^V12^* with *HmlΔ*-GeneSwitch both produced viable progeny when RU486 was supplied continuously from embryonic stages onwards. Representative images of flies at 6 days post-eclosion that are expressing *Stinger* only (‘Control’), *bax* and *Stinger*, or *ras85D^V12^* and *Stinger* (left to right). (B) Count of *HmlΔ*-positive cells at 6 days post-eclosion using the Find Maxima tool in Fiji identified an increase in *HmlΔ*-positive cells in flies where *HmlΔ*-*GeneSwitch* was driving *ras85D^V12^* and a strong reduction of labelled cells when *bax* was driven. A two-way ANOVA with *post hoc* Tukey HSD corrections was used to assess statistical significance relative to controls, as well as obtain 95% confidence intervals for the effect size of each transgene. ****P<0.0001; *n*≥9 flies per condition. The exact genotypes used in this figure were: ‘Control’=*w^1118^*;+;*UAS-Stinger*/*HmlΔ-GeneSwitch*; ‘*bax*’=*w^1118^*;+/*UAS-bax*;*UAS-Stinger*/*HmlΔ-GeneSwitch*; ‘*ras*’=*w^1118^*;+/*UAS*-*ras85D^V12^*;*UAS-Stinger*/*HmlΔ-GeneSwitch*.

*HmlΔ-Gal4* is a constitutive driver and leads to the expression in differentiated blood cells from larval stages onwards. This approach, however, requires the crossing of different genetic lines that are unlikely to be genetically identical. Conditional drivers have the benefit of controlling fully for genotype, therefore we generated a *HmlΔ*-*GeneSwitch* transgenic line. The *HmlΔ*-*GeneSwitch* construct we made resulted in strong expression of a *UAS-Stinger* transgene by 1 day post-eclosion, when activated by RU486 throughout development (Fig. S1A). Under both ablated and expanded conditions (*HmlΔ-GeneSwitch* driving *UAS-bax* and *UAS-ras85D^V12^*, respectively, with RU486 provided throughout development and continuing post-eclosion), a modulation of cell number was detected with both transgenes 6 days post-eclosion (Fig. 2; ablation data for 1 day post-eclosion shown in Fig. S1B), demonstrating that *HmlΔ*-*GeneSwitch* can fully reproduce results obtained via *HmlΔ-Gal4* (Fig. 1). Note that our ability to visualise cells using *UAS-Stinger* requires induction of this system, thus we only visualise cells that have an active *HmlΔ* promoter.

Bleeding of larvae and adults allows measurement of total numbers of blood cells (including cells not labelled by *UAS-Stinger*), with the caveat that it does not capture cells tightly integrated into tissue or strongly adhered, which may therefore be harder to release during dissections. Despite this limitation, imaging of L3 larvae ahead of dissection demonstrated a strong enhancement of numbers of *Hml*-labelled cells compared with levels in controls upon constitutive expression of *ras85D^V12^* via *HmlΔ-Gal4*, whereas almost no cells were visible on expression of *bax* (Fig. S2A–C). However, larval bleeds from the same genotypes revealed significant increases in total haemocyte numbers via both strategies (Fig. S2D–G). Very few Stinger-positive cells survived upon expression of *bax* and many of those cells exhibited a lamellocyte-like morphology, whereas almost all cells present upon *ras85D^V12^* expression were Stinger-positive (Fig. S2E,F).

Bleeding of adult flies revealed that a significant difference was only achieved for flies in which *bax* was driven using constitutive *HmlΔ-Gal4*, with only a mild reduction in haemocytes observed (and then only in females). Furthermore, driving *ras85D^V12^* did not significantly expand numbers of bled cells (Fig. S2H,I). As per larvae, many cells present in adult bleeds upon expression of *bax* exhibited lamellocyte-like morphologies (data not shown).

**Fig. 3.**
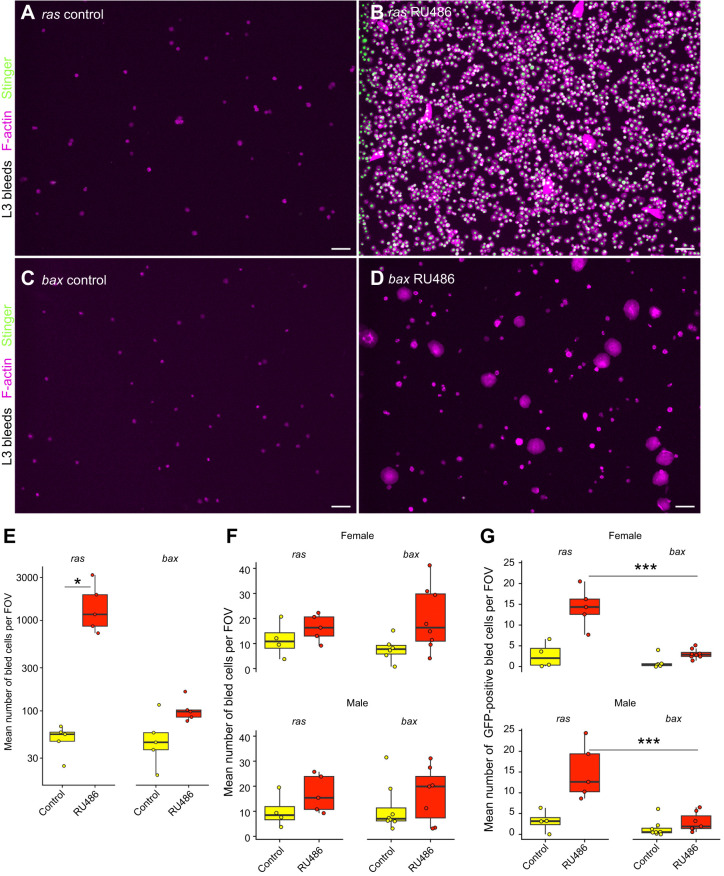
**Cell counts and representative images of cells bled from larvae and adults in which Stinger and either *ras85D^V12^* or *bax* were driven by *HmlΔ-GeneSwitch*.** (A–D) Haemocytes were bled from L3 larvae containing *HmlΔ-GS* and *UAS-Stinger*, and either *UAS-ras85D^V12^* (constitutively active variant, A,B) or *UAS-bax* (C,D). Larvae were grown in the absence of RU486 as a control (*ras* control/*bax* control; A,C), or the presence of RU486 to induce expression (*ras* RU486/*bax* RU486; B,D). Images show F-actin (magenta) and Stinger (green) fluorescence; scale bars: 50 μm. (E) Box plots showing mean number of cells per field of view (FOV) for L3 larval bleeds with and without induction via *HmlΔ-GS* and RU486; *n*=5 larvae for each condition. (F,G) Box plots showing mean number of cells (F) and mean number of GFP-positive cells (i.e. marked via expression from *UAS-stinger*, G), per FOV, per adult dissection; n.b., two flies dissected per well (*n*≥4 for each condition with individual data points plotted). For all bleed data, each point represents the total cells bled per FOV from 2 flies or 1 larva. Statistical significance was calculated using ANOVAs, with pairwise comparisons assessed using Tukey's HSD *post hoc* testing; *P≤0.05 and *** *P*≤0.001. All genotypes are as follows: *w^1118^;+/UAS-ras85D^V12^;HmlΔ-GAL4/UAS-Stinger* (*ras* control/*ras* RU486)*, w^1118^;+/UAS-bax;hmlΔ-GAL4/UAS-Stinger* (*bax* control/*bax* RU486).

Conducting the equivalent experiments using *HmlΔ*-*GeneSwitch*, for which the food was supplemented with RU486 throughout development, revealed very similar results to the constitutive approaches (Fig. 3): in *HmlΔ*-*GeneSwitch* larvae driving *ras85D^V12^*, the number of cells was increased more than 30-fold, whereas larvae in which *bax* was driven showed a trend towards an increase in cell number, although this did not achieve statistical significance (*P*=0.06; Fig. 3A–E).

In adults where transgenes were driven by *HmlΔ*-*GeneSwitch*, no statistically significant changes in total cell numbers were observed (Fig. 3F), although the number of *Hml*-positive cells (interpreted from GFP labelling) was significantly increased in flies where *ras85D^V12^* was driven by *HmlΔ*-*GeneSwitch*, relative to those in which *bax* was driven (Fig. 3G).

These data suggest the existence of limitations or feedback mechanisms that control numbers of haemocytes that can be present within adults, given that, despite dramatic increases in cell numbers in larvae, the effect on numbers of haemocytes in adults is less pronounced upon expression of *ras85D^V12^*. Potentially, rather than modulating the number of total haemocytes, our genetic tools may in fact specifically modulate numbers of *Hml*-positive cells.

### Manipulating haemocyte cell numbers does not affect adult lifespan

**Fig. 4.**
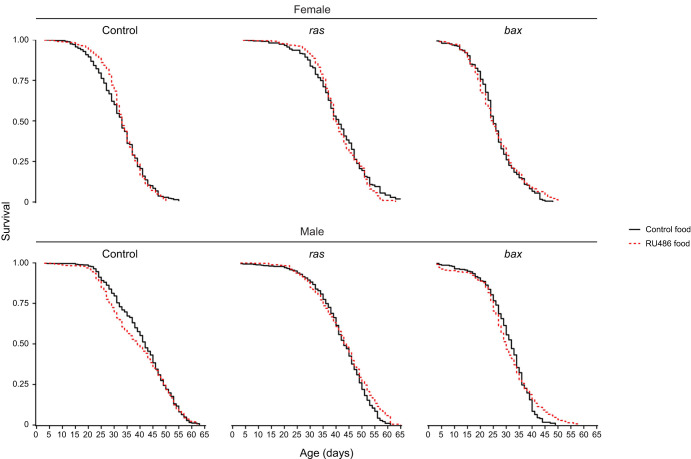
**Neither ablation nor expansion of *HmlΔ*-positive cells significantly affects lifespan using the conditional *HmlΔ*-*GeneSwitch* driver.** *HmlΔ*-*GeneSwitch* was activated from larval stages using food supplemented with RU486, and following eclosion flies were mated for 48 h. Subsequently flies were sorted into lifespan cages, for which new food was provided every 48 h and the number of dead flies was counted. For ‘Control’ flies, *HmlΔ*-*GeneSwitch* drove only the *UAS*-*Stinger* transgene, whereas for ‘bax’ and ‘ras’ flies, either *UAS*-*bax* or *UAS*-*ras85D^V12^* were driven in addition to *UAS*-*Stinger*. The exact genotypes used above were as follows: ‘Control’=*w^1118^*;+;*UAS*-*Stinger*/*HmlΔ*-*GeneSwitch*; ‘ras’=*w^1118^*;+/*UAS*-*ras85D^V12^*;*UAS*-*Stinger*/*HmlΔ*-*GeneSwitch*; ‘bax’=*w^1118^*;+/*UAS*-*bax*;UAS-*Stinger*/*HmlΔ*-*GeneSwitch*. Details of the statistical analysis for this figure can be found in Table S1A.

Considering that immune function is central to ageing (Stanley et al., 2023), we wanted to investigate whether manipulating the haemocyte population would impact longevity. Upon testing this with large sample sizes under highly controlled conditions, we observed alterations to lifespan upon both *Hml*-positive cell expansion and ablation using constitutive expression of *ras85D^V12^* or *bax* via *HmlΔ-Gal4* (Fig. S3A). However, this effect was not replicated when repeating this using the conditional *HmlΔ-GeneSwitch* system and continuous application of RU486, despite ensuring that all other aspects of the experiment were identical (Fig. 4; statistics shown in Table S1A). This strongly suggests that small genetic differences between the *UAS* lines explained the difference in longevity observed via the constitutive *HmlΔ-Gal4* approach.

Indeed, similarly, *bax*, *ras85D^V12^* and control crosses with the conditional driver showed the same lifespan differences; the *bax* crosses lived for the shortest times, whereas the *ras85D^V12^* crosses lived longest, but with no additional modulation by driving the ablation or expansion using RU486 (Fig. 4). Additionally, to test this hypothesis further, we crossed the same *bax*, *ras85D^V12^* and control lines to a *yw* background and found similar lifespan differences to those observed when using the constitutive *HmlΔ*-*Gal4* driver, particularly for the reduced lifespan observed with *bax* (Fig. S3B). These observations fit with the idea that lifespan is a highly polygenic trait and that the accumulation of deleterious mutations through drift can strongly determine lifespan (Cui et al., 2019), underscoring the importance of conditional driver systems, especially when studying ageing (Ford and Tower, 2005). It remains possible that stronger enhancement of haemocyte numbers could impact lifespan, whereas the non-*Hml* positive haemocytes that appear expanded upon expression of *bax* could mask effects on lifespan linked to compromised immunity.

**Fig. 5.**
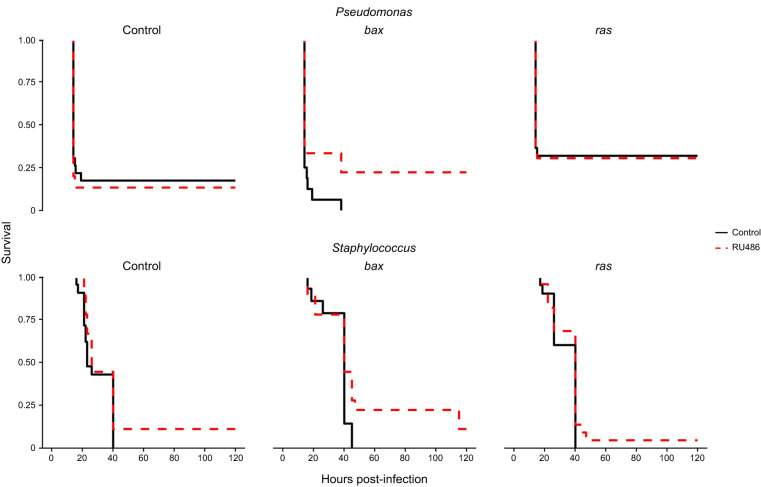
**Conditional manipulation of *Hml*-positive number does not significantly affect post-infection survival with *Pseudomonas entomophila* or *Staphylococcus aureus* when using the conditional *HmlΔ*-*GeneSwitch*.** Female flies containing *HmlΔ*-*GeneSwitch* and the transgenes *UAS*-*bax*, *UAS*-*ras85D^V12^* or *UAS*-*Stinger* (the control, all flies tested also possessed this transgene) were either activated or not in female flies by the presence of RU486 throughout development and infected with *Pseudomonas entomophila* (top) or *Staphylococcus aureus* (bottom) at 5 days post-eclosion. No statistically significant modulation of post-infection survival was observed upon infection with either bacterium (statistics shown in Table S1B). Infected flies were checked for death every 30 min from approximately 18 h post-infection until approximately 25 h post-infection, and less frequently after this point. Experiments were terminated 120 h post-infection. No flies ‘infected’ with a sham infection (water) died over the 120 h time course (not shown on plot). *N*≥14 for each treatment group. The exact genotypes used above were as follows: ‘Control’=*w^1118^*;+;*UAS*-*Stinger*/*HmlΔ*-*GeneSwitch*; ‘bax’=*w^1118^*;+/*UAS*-*bax*;*UAS*-*Stinger*/*HmlΔ*-*GeneSwitch*; ‘ras’=*w^1118^*;+/*UAS*-*ras85D^V12^*;*UAS*-*Stinger*/*HmlΔ*-*GeneSwitch*.

We investigated whether *Hml*-positive cell number affects survival from a pathogen infection, as previously reported using constitutive drivers (Defaye et al., 2009; Charroux and Royet, 2009) and including a small number of experiments using a temperature-sensitive conditional system (Defaye et al., 2009). Immune challenges with two commonly used pathogens (*Pseudomonas entomophila* or *Staphylococcus aureus*) to study immunity in the fly were not affected by conditional *Hml*-positive cell ablation or expansion (Fig. 5; statistics shown in Table S1B and dose optimisation shown in Fig. S4). These results therefore imply that neither lifespan nor post-infection survival are severely impacted by modulating the number of *Hml*-positive cells as performed here. Again, more significant expansion of *Hml*-positive cells or the presence of increased numbers of *Hml*-negative haemocytes could obscure immune phenotypes in these assays.

### Modulation of *Hml*-positive cell number using *HmlΔ*-*GeneSwitch* affects the haemocyte-dependent phenotype of *tuSz^1^*

**Fig. 6.**
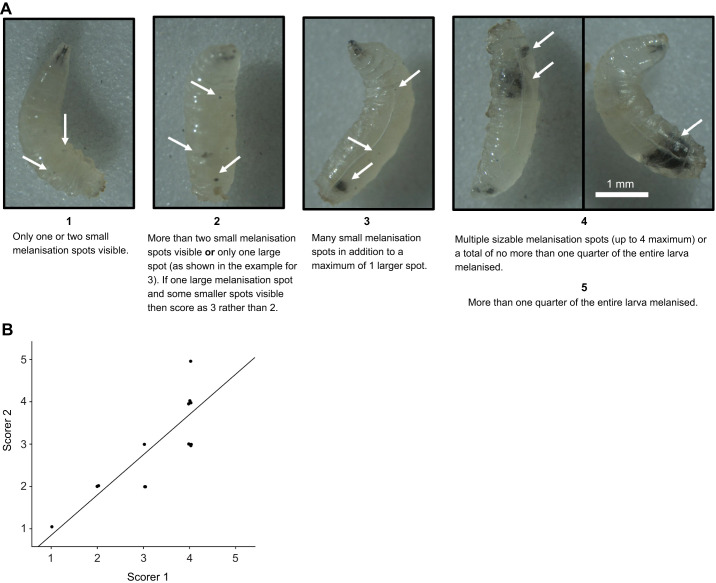
**A novel semi-quantitative larval melanisation scoring system.** (A) A novel melanisation scoring system was devised, for which L3 larvae showing signs of melanisation are picked and scored from 1 to 5 based on the displayed criteria. Although every male larva displayed some level of melanisation in the experiments undertaken here, the scoring system could conceivably be extended to include a 0 score as well, to signify absolutely no excessive melanisation at all, if required. Examples of each score are also shown, with white arrows indicating notable features of each score. Two examples of larvae, both of which would be scored as 4 are shown. In the experiments undertaken here, no larvae were severe enough to be given a score of 5, but the criterion for a score of 5 is also shown. (B) A blinded correlation of scores given to 15 larvae by two scorers using the melanisation scoring system. A Spearman correlation test resulted in ρ=0.856 and *P*=4.72×10^−5^, signifying a good correlation between the two independent sets of scores given. A small random value (−0.05 to 0.05) is added to each score on the correlation plot, for the purposes of illustrating the individual points present, but these values were not used for the statistical analysis. All larvae used to produce this larval melanisation scoring system contained the *tuSz^1^* X chromosome mutation.

**Fig. 7.**
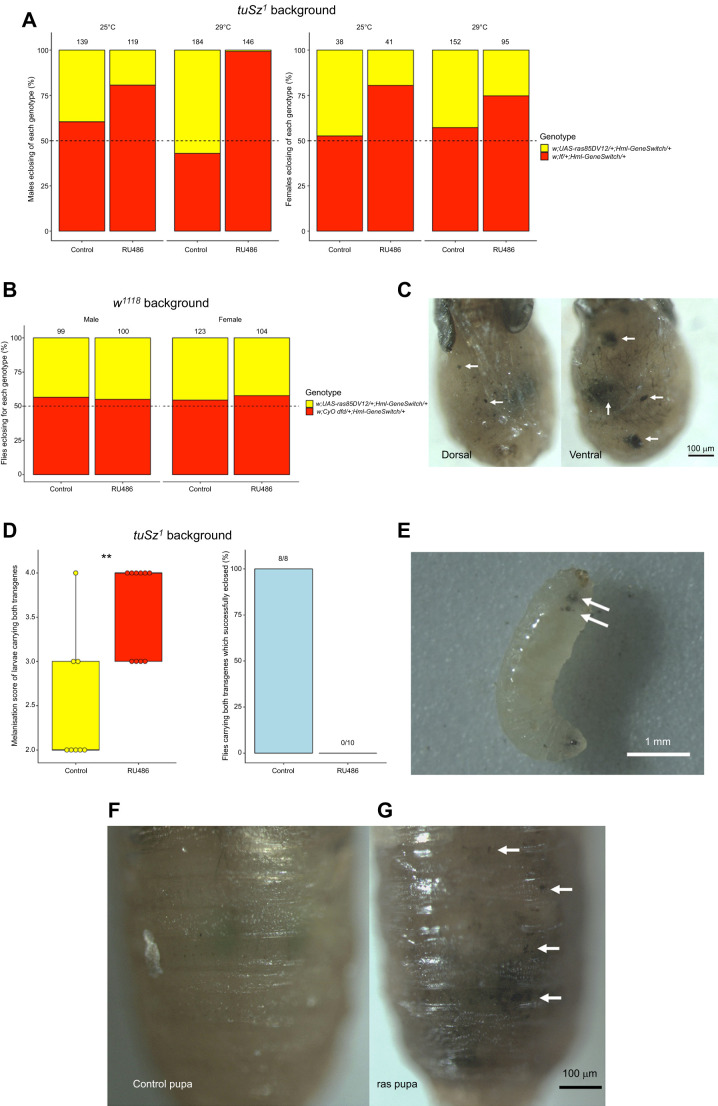
**Driving *UAS*-*ras85D^V12^* with *HmlΔ*-*GeneSwitch* causes pupal lethality as a result of excessive melanotic tissue.** (A) Far fewer male flies eclose with both the *UAS*-*ras85D^V12^* and *HmlΔ*-*GeneSwitch* transgenes when grown at 29°C in the presence of RU486 than female flies in any condition, or males in the absence of RU486 or grown at 25°C. The *If* marker was used to identify whether flies enclosed with both transgenes or with only *HmlΔ*-*GeneSwitch*, and therefore the expected ratio of each genotype was 50% (indicated by the dotted line). The numbers above each bar signify how many total eclosed flies were counted per condition. The cross to generate these flies was as follows: w*^tuSz1^* (female containing the *tuSz^1^* mutation) X *w^1118^*;*If*/*UAS*-*ras85D^V12^*;*HmlΔ*-*GeneSwitch* (male). (B) Driving *UAS*-*ras85D^V12^* with *HmlΔ*-*GeneSwitch* in the absence of the *tuSz^1^* mutation did not result in developmental lethality at 29°C in either males or females. The dotted line indicated the expected ratio of flies carrying both the *HmlΔ*-*GeneSwitch* and *UAS*-*ras85D^V12^* trangenes, which was 50%. The flies crossed here to test developmental lethality were of the following genotypes: *w^1118^*;;*HmlΔ*-*GeneSwitch* (female) X *w^1118^*;*UAS*-*ras85D^V12^*/*CyO dfd* (male). (C) Male flies of the genotype *w^tuSz1^*;*UAS*-*ras85D^V12^*;*HmlΔ*-*GeneSwitch* grown at 29°C in the presence of RU486 arrested at a late pupal stage. Upon removal of the puparium, significant levels of melanotic tissue in the fat body were observed (white arrows). (D) Male L3 larvae from the same experiment were scored for levels of melanisation using the novel scoring system (Fig. 6). These larvae were followed individually throughout development so as to confirm their genotype upon eclosion. Of the flies with both *UAS*-*ras85D^V12^* and *HmlΔ*-*GeneSwitch*, flies fed food containing RU486 showed a statistically significant increase in melanisation relative to those fed a control diet. A two-sided Student's *t*-test was used to assess statistical significance, for which ** signifies *P*-value<0.01. 27 male larvae on a control diet and 25 male larvae on an RU486-containing diet were originally picked from each condition, of which 8 control diet-fed larvae and 10 RU486-fed harboured both *UAS*-*ras85D^V12^* and *HmlΔ*-*GeneSwitch*. All 8 of the control-fed larvae successfully pupated and eclosed, whereas none of the 10 RU486-fed larvae eclosed, and instead aborted development at approximately the P12 pupal stage, as previously observed. (E) Example larva of the genotype *w^tuSz1^*;*UAS*-*ras85D^V12^*/+;*HmlΔ*-*GeneSwitch*/+ with clear excess melanisation visible, highlighted by white arrows. (F,G) Pupae in which the *Hml*-positive cell population had been expanded using *HmlΔ-GeneSwitch* (*ras* pupa, G) showed high levels of excessive melanisation compared with control pupa (F), which was clear even through the puparium. White arrows indicate clear melanotic spots in the example *ras* pupa.

Using our novel *HmlΔ-GeneSwitch* driver, we were able to change the number of *Hml*-labelled cells, but this was not associated with infection or lifespan phenotypes. Therefore, we wanted to test another known haemocyte-related function upon manipulation of their numbers, as a positive control. To do this, we used the *tuSz^1^* mutant, which has a mutation on the X chromosome, 35 bp upstream of the *GCS1* transcription start site. This mutation results in a loss of self-tolerance in the posterior fat body and a haemocyte-driven self-encapsulation phenotype that is temperature dependent (Mortimer et al., 2021). We developed a novel scoring system in order to quantify this self-encapsulation phenotype (Fig. 6). We hypothesised that expansion of the haemocyte cell population would lead to increased targeting of misrecognised ‘self’ tissue in the presence of the *tuSz^1^* mutation (Mortimer et al., 2021). Indeed, we observed developmental lethality when *ras85D^V12^* was overexpressed using *HmlΔ*-*GeneSwitch*, with lethality stronger at 29°C (the temperature at which the melanisation phenotype presents itself in control flies). In males at the higher temperature, where the *tuSz^1^* mutation cannot be rescued by a second copy of the X chromosome, near-complete developmental lethality was observed (Fig. 7A and Table 2); only a single fly eclosed at 29°C where *UAS*-*ras85D^V12^* had been activated by *HmlΔ*-*GeneSwitch* and this fly died within 3 days of eclosion. Flies that overexpressed *ras85D^V12^* alone at 29°C, without the *tuSz^1^* mutation, showed no developmental lethality (Fig. 7B and Table 2). Flies overexpressing *ras85D^V12^* in the same genetic background but at 25°C also showed no developmental lethality (Fig. S5A and Table 2), similarly, the 25°C RU486-driven flies showed no significant difference to flies grown at 25°C where *ras85D^V12^* was driven by the constitutive *HmlΔ*-*Gal4* driver (Table 2, counts from Fig. 1B; these flies were female only so as to match the constitutive counts). This therefore suggested that there is no difference in effect on lethality of *UAS*-*ras85D^V12^* between the constitutive and conditional *HmlΔ* drivers. We also repeated these experiments using flies carrying the *UAS-bax* transgene to test whether reduction of the *Hml*-positive cell population using *UAS-bax* driven by *HmlΔ-GeneSwitch* affected development and ability to eclose in the presence of the *tuSz^1^* mutation or on a *w^1118^* background. In this experiment, we observed no successful eclosers for either sex in 29°C RU486 conditions in the *tuSz^1^* background, although flies did successfully eclose in the *w^1118^* background. As with the equivalent *UAS-ras85D^V12^* experiments, activation of the *HmlΔ-GeneSwitch* driver through supplementation of RU486 reduced eclosion rates at both temperatures in the context of the *tuSz^1^* background, although unlike the *ras85D^V12^*-carrying flies, RU486 supplementation to *UAS-bax* flies also reduced eclosion rates in the context of the *w^1118^* genetic background. Furthermore, again as found with the *ras85D^V12^* experiment, the strength of effect of *HmlΔ-GeneSwitch* driving *UAS-bax* did not significantly differ from that of *HmlΔ-Gal4* driving the same transgene (Fig. S5B,C and Table 2).

**Table 2.** χ**^2^ statistics for each eclosion comparison**

Comparison	Genetic background	Transgene	χ^2^ statistic	*P*-value
Male 25°C RU486 vs control	*tuSz^1^*	*ras85D^V12^*	12.45 (d.f.=1)	0.00042
Female 25°C RU486 vs control	*tuSz^1^*	*ras85D^V12^*	6.93 (d.f.=1)	0.0085
Male 29°C RU486 vs control	*tuSz^1^*	*ras85D^V12^*	118.68 (d.f.=1)	<0.00001
Female 29°C RU486 vs control	*tuSz^1^*	*ras85D^V12^*	7.77 (d.f.=1)	0.0053
Male 29°C RU486 vs control	*w^1118^*	*ras85D^V12^*	0.049 (d.f.=1)	0.82
Female 29°C RU486 vs control	*w^1118^*	*ras85D^V12^*	0.24 (d.f.=1)	0.63
Male 25°C RU486 vs control	*w^1118^*	*ras85D^V12^*	0.001 (d.f.=1)	0.97
Female 25°C RU486 vs control	*w^1118^*	*ras85D^V12^*	0.0009 (d.f.=1)	0.98
Female 25°C *HmlΔ-GeneSwitch* RU486 vs *HmlΔ-Gal4*	*w^1118^*	*ras85D^V12^*	0.84 (d.f.=1)	0.36
Male 25°C RU486 vs control	*tuSz^1^*	*bax*	15.099 (d.f.=3)	0.0017
Female 25°C RU486 vs control	*tuSz^1^*	*bax*	12.19 (d.f.=3)	0.0068
Male 29°C RU486 vs control	*tuSz^1^*	*bax*	33.25 (d.f.=3)	<0.00001
Female 29°C RU486 vs control	*tuSz^1^*	*bax*	67.018 (d.f.=3)	<0.00001
Male 29°C RU486 vs control	*w^1118^*	*bax*	24.15 (d.f.=1)	<0.00001
Female 29°C RU486 vs control	*w^1118^*	*bax*	53.85 (d.f.=1)	<0.00001
Male 25°C RU486 vs control	*w^1118^*	*bax*	5.43 (d.f.=1)	0.02
Female 25°C RU486 vs control	*w^1118^*	*bax*	22.0 (d.f.=1)	<0.00001
Female 25°C *HmlΔ-GeneSwitch* RU486 vs *HmlΔ-Gal4*	*w^1118^*	*bax*	1.70 (d.f.=1)	0.19

The degrees of freedom (d.f.) used for each comparison are shown in the χ^2^ statistic column. For comparisons of *HmlΔ-GeneSwitch* RU486 against *HmlΔ-Gal4*, counts from Fig. 1B were used as the comparison.

Melanisation is most easily detected at larval stages (Fig. 7E), but it was clear that the *ras85D^V12^* flies that failed to eclose showed excessive melanisation and this could be observed even without removal of the pupal case (Fig. 7F,G). Removal of the puparium revealed that these pupal-lethal flies consistently died at approximately the P12 pupal stage (Bainbridge and Bownes, 1981) and showed significant levels of excessively melanised tissue in the fat body, which was likely responsible for the observed developmental lethality (Fig. 7C).

Conditional expansion of the *Hml*-positive cell population also increased melanisation during larval stages. RU486-fed larvae showed significantly higher melanisation scores relative to the control diet-fed larvae (Fig. 5D,E). For this purpose, we single-housed larvae to determine their genotype retrospectively (using *If* as a marker to genotype flies; see Materials and Methods). As in our earlier experiment, no male larvae containing both *HmlΔ*-*GeneSwitch* and *UAS*-*ras85D^V12^* that had been fed on RU486-containing media successfully developed and eclosed at 29°C. Furthermore, the genotypic proportions of pupae which eclosed were approximately the same as observed when larvae were not kept in individual vials, ruling out the possibility that the lethality phenotype was a result of competition between larvae of different genotypes (Fig. 7A compared with Fig. 7D). Overall, these results demonstrate that although no lifespan or infection survival phenotypes are observed upon conditional *Hml*-positive cell number modulation, manipulation of *Hml*-positive cell number using *HmlΔ*-*GeneSwitch* can produce strong functional haemocyte-specific effects.

## DISCUSSION

Our work demonstrates that a constitutive haemocyte-specific driver (*HmlΔ*-*Gal4*) is able to not only reduce *Hml*-positive cell numbers, but also expand the population, via expression of *bax* and *ras85D^V12^* transgenes, respectively (Fig. 1A,B). Additionally, a conditional *HmlΔ*-*GeneSwitch* driver produced comparable effects (Fig. 2). However, bleeding haemocytes from adults and larvae suggested that we were not affecting all immune cells and that compensatory mechanisms can impact the overall population *in vivo* (Fig. 3 and Fig. S2). We confirmed that *Hml*-positive cell number expansion modulated a known haemocyte-dependent phenotype (self-encapsulation; Fig. 7). However, manipulating *Hml*-positive cell populations had no effect on lifespan (Fig. 4) or survival post-immune challenge with two different pathogens (Fig. 5), in spite of a lifespan modulation being initially observed via a constitutive *HmlΔ*-*Gal4*-mediated approach (Fig. S3A). No difference was observed between the strength of the constitutive and conditional *HmlΔ* drivers in terms of lethality as a result of expanding the haemocyte population (comparison between counts in Fig. 1C and Fig. S5A), thus reducing the likelihood that no effect was observed using our conditional *HmlΔ* as a result of it being weaker than *HmlΔ*-*Gal4*. These negative findings on longevity and especially post-infection survival contrast to a number of studies in the literature. There are several reasons for these differences that we discuss below, with the foremost reason being that other *Hml*-negative immune cells likely replace *Hml*-positive haemocytes on ablation with *bax*.

The literature suggests that haemocyte ablation should affect survival following pathogen exposure (Shia et al., 2009; Charroux and Royet, 2009; Arefin et al., 2015; Defaye et al., 2009). However, the severe pupal lethality and increased self-encapsulation phenotype observed in flies in which the *Hml*-positive population was expanded in the context of the *tuSz^1^* mutation suggests that increasing *Hml*-positive cell number really does upregulate haemocyte activity. Note, however, that we cannot distinguish cell number from other possible pleiotropic effects of expressing constitutive-active Ras, although Ramond et al. (2020) found limited evidence of activation of stress-associated pathways via overlapping approaches to manipulate haemocytes; similar considerations hold for *bax* as an experimental strategy. Regardless, it is surprising that we did not observe other adult phenotypes. Although the infected flies in our experiments died within a similar timeframe as prior experiments, it is possible that the dose of the immune challenge masks any modulating effect from haemocytes. Potentially therefore, haemocyte number may be critical in the face of infection only at certain levels of infection. Another explanation is that there is compensation within the population of cells. It is also possible that the *Hml*-negative cells are sufficient to protect against infection and that the expansion in adults via *ras* expression does not reach a threshold to exert harmful or beneficial effects. It is also now known that haemocytes communicate with cross-talk between these cells and other tissues (Yang and Hultmark, 2016; Green et al., 2018). As such, it remains possible that both ablation and expansion of the *Hml*-positive population functionally does little, except if cells are falsely attracted to ‘self’. Perhaps, the expanded cells are differentiated such that functioning is increased in recognition of ‘self’ rather than any other functions. Indeed, a recent study used the pro-apoptotic genes *hid* and *reaper* to ablate *Hml*-positive haemocytes and found an increase in *Hml*-negative plasmatocytes, in line with our data and interpretation (Shin et al., 2020) and expansion of lamellocyte-like cells on ablation was also seen by Arefin et al. (2015). These *Hml*-negative haemocytes were shown to express *Pxn*, a classical haemocyte marker gene (Evans et al., 2014), despite the lack of *Hml*, and therefore it would be beneficial in future work to test whether our *Hml-*negative haemocyte population also expresses *Pxn*, in order to fully confirm that our results here identified the same *Hml*-negative population. The expansion of lamellocyte-like cells observed by both us and Arefin and colleagues is likely a result of lamellocytes lacking *Hml* expression (Goto et al., 2003), so enabling this lineage of haemocytes to dominate in *Hml*-positive cell ablating conditions, in part at least as they are thus able to escape expression of *bax*. A separate study also concluded that depletion of *Hml*-positive cells was insufficient to generate flies completely lacking haemocytes, as *Hml*-negative haemocytes remained unaffected (Boulet et al., 2021). Their study also suggested that depletion of *Hml*-positive immune cells may affect the gene expression of the remaining immune cells, in line with our own work that has previously shown exposure to apoptosis can impact subpopulations at other stages of development (Coates et al., 2021; Brooks et al., 2024). These observations perhaps fit with our observation of an increase in lamellocyte-like cells. Regardless, it is essential that future studies further investigate methods of haemocyte depletion without limiting themselves exclusively to *Hml* drivers. Furthermore, the populations of *Hml*-negative immune cells which remain following *Hml*-positive cell depletion offer an explanation for the lack of infection phenotype in flies where the haemocyte population has been ‘ablated’ or ‘expanded'. These possibilities will prove a fruitful paradigm to study immunology in the fly; an emerging theme in this area of research are functional differences within the plasmatocyte lineage of haemocytes (Shin et al., 2020; Coates et al., 2021).

It is interesting that *ras85D^V12^* dramatically increased the total cell population in larvae, but not in adults. This is an intriguing discovery, which suggests that potentially a feedback mechanism exists in adult flies to restrict numbers of *Hml*-positive cells, which are not present or as active in larvae. This is a discovery that is not only relevant for fly biology, but also for potential use as a cancer model. Feedback mechanisms that regulate the number of immune cells are highly relevant to understanding immunoproliferative disorders such as leukaemia or lymphoma (Connors et al., 2020; Tebbi, 2021). Of further relevance, recent work from the Giangrande lab revealed early wave haemocytes can impact later blood cell development via extracellular matrix deposition, demonstrating potential feedback mechanisms are relevant in the fly (Monticelli et al., 2024).

Blood cell numbers are tightly controlled in higher organisms at the level of generation and release from the bone marrow, with clearance from circulation via entry into tissues (Scheiermann et al., 2015). Most commonly, blood cells are ultimately removed via apoptotic cell clearance in organs such as the liver and spleen. The retraction of blood cell numbers we have observed appears to occur between late larval stages and eclosion as adults. Given that no further proliferation has been identified during pupal stages and that haemocytes are released from the lymph gland after the end of larval stages, it is unlikely that feedback mechanisms would supress proliferation or release, as this would be too late to impact cell numbers. Cell death, most likely apoptosis, is thus the prime candidate here. More typically, Ras gain-of-function mutations are associated with a poor prognosis in cancer and increased rates of proliferation/cell survival, including in *Drosophila* (e.g. Gafuik and Steller, 2011). However, some studies suggest Ras activation can sensitise some tumour cells to chemotherapy or lead to apoptosis (Overmeyer and Maltese, 2011). Therefore, one possibility might be an intrinsic mechanism whereby excessive levels of Ras activity eventually drive apoptosis.

Alternatively, a more systemic mechanism might be at play, potentially orthologous to control of vertebrate neutrophil numbers: here clearance of apoptotic neutrophils inhibits IL-23 release; since IL-23 would normally stimulate T cell-mediated IL-17 release and drive subsequent elevation of G-CSF, this limits further blood cell production (Stark et al., 2005). Other hypotheses might include insufficient levels of pro-survival factors like TGF beta or PDGF/VEGF-related ligands to maintain this size of population, given the previous roles for these ligands in mediating haemocyte survival (Bruckner et al., 2004; Makhijani et al., 2017). What is clear is that elevated numbers of haemocytes can strongly perturb fly physiology with large expansions demonstrated to lead to lethality under some conditions (Ramond et al., 2020) or ‘selfishly’ reduce access of non-immune organs to nutrients (Bajgar and Dolezal, 2018). Thus, there is a strong rationale for negative regulation of cell numbers to maintain organismal health. Complex feedback networks regulate haemocyte proliferation, differentiation and release in flies (see Bannerjee et al., 2019 for review; Madhwal et al., 2020), though these pathways are more associated with promotion of cell survival than as drivers of immune cell apoptosis, so this remains an exciting avenue for future experimentation.

Flies in which haemocytes are ablated display melanotic spots, purportedly as a result of the lamellocyte population expanding upon *HmlΔ*-positive cell ablation (Defaye et al., 2009; Arefin et al., 2015). We observed similar melanotic spots when we ablated the *Hml*-positive cell population with the constitutive *HmlΔ*-*Gal4* driver (data not shown). Furthermore, we observed an increase in melanotic spots in the context of the *tuSz^1^* mutation when the haemocyte population was expanded. It is not clear how these two melanotic phenotypes are related. The ablation-related spots identified by previous work were found across the whole larval body (both posterior and anterior), whereas the melanisation generated by the *tuSz^1^* mutation is found only in the larval posterior (Mortimer et al., 2021). Furthermore, the *tuSz^1^*-mediated melanisation persists into adult flies, whereas the melanotic phenotype previously identified when haemocytes were ablated is only mentioned in relation to larvae (Defaye et al., 2009; Arefin et al., 2015). Although these phenotypes appear superficially similar, they may in fact originate via different mechanisms.

The flies utilised in this body of work were not grown in a sterile environment, and so our expectation was that modulation of haemocyte number would affect lifespan. Prior studies have shown other adult phenotypes for haemocyte ablation, and perhaps if we could ablate more or all *Hml*-positive cells a phenotype would be observed, although very few *Hml*-positive cells were left in flies in which we ablated this cell population. Indeed, the *Hml*^P2A^ driver is suggested to be stronger and active in a larger fraction of the haemocyte population (Stephenson et al., 2022). However, previous uses of the *HmlΔ* promoter have shown clear immune phenotypes upon challenge with pathogens (Shia et al., 2009; Charroux and Royet, 2009; Arefin et al., 2015; Defaye et al., 2009; Ayyaz et al., 2015). The only obvious difference in the approach we have used here compared with previous constitutive (or temperature-sensitive conditional) approaches is that our driver only starts to become active upon the first instance of larval feeding, as the chemical required to activate the GeneSwitch construct was in our experiments administered via the media. This should have a limited impact, considering that *Hml* is considered to be active only from early larval stages (Charroux and Royet, 2009); however, there is the potential for a narrow window during which *Hml* becomes active, so promoting transcription of the GeneSwitch construct, but before RU486 is ingested via first larval feeding. In essence this means that *HmlΔ*-positive cells may be present for a short period in our conditional haemocyte manipulation system where they would not be present when using a constitutive driver. Similarly, levels of RU486-dependent activation may drop during pupal stages when active feeding has ceased. Overall, it seems unlikely that all previously documented immune phenotypes of ablating haemocytes are the result of ablation at this very early larval or pre-larval stage, and that any future ablation has no immune effect, but it cannot be ruled out.

Overall, our work demonstrates that a conditional *HmlΔ*-*GeneSwitch* driver is able to drive not only ablation of *Hml*-positive cells, but also expansion of this population in flies. However, modulating haemocyte numbers conditionally did not affect longevity, in contrast to our observations when using a constitutive driver, nor did it affect immunity. This leads to our conclusion that rare alleles that are fixed at random but are determinative of lifespan likely explain these differences. Lifespan is thus a phenotype in which it is especially important to control for genetic background, as has been argued before (Ford and Tower, 2005). Our study therefore illustrates the utility of the *HmlΔ*-*GeneSwitch* driver to understand macrophage behaviour in the fly model. The work also suggests powerful mechanisms of regulation for overall numbers of immune cells in the fly that may not always be trivial to overcome experimentally. In this process, we identified potentially important but unknown mechanisms that regulate total immune cell numbers, for which conditional and especially temporal manipulation (Simons et al., 2019 preprint) using GeneSwitch provides a promising experimental paradigm.

## Supplementary Material

10.1242/jexbio.249649_sup1 Supplementary information
