# Supplementary material for: Expansion of Drosophila haemocytes using a conditional GeneSwitch driver affects larval haemocyte function, but does not modulate adult lifespan or survival after severe infection
Source: J Exp Biol. 2025 May 6;228(9):jeb249649. doi: 10.1242/jeb.249649 (PMC12079669; doi:10.1242/jeb.249649)
Supplement: Supplementary information [file jexbio-228-249649-s1.pdf]

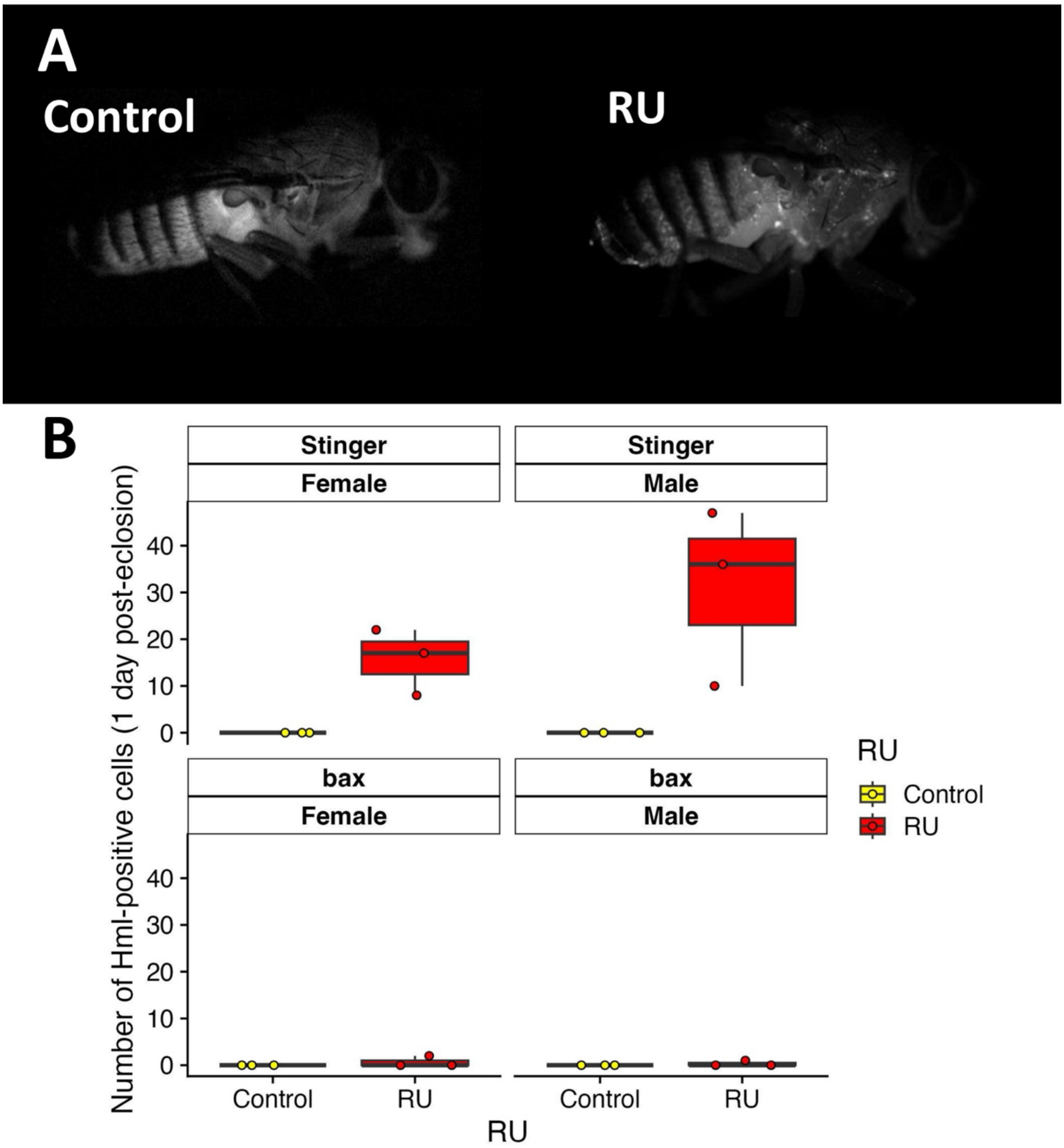

Fig. S1. The *HmlΔ-GeneSwitch* construct can ablate hemocytes at 1-day post-eclosion when driving *bax*, when flies are fed RU throughout development. (**A**) The *HmlΔ-GeneSwitch* construct drives the *UAS-Stinger* transgene, observed in fluorescence at 1-day post-eclosion when food was supplemented with RU during development. No fluorescence was detected in controls not fed RU, suggesting there is limited or no leaky expression from these constructs. The flies used here were of the following genotype: w^{1118};+;*HmlΔ-GeneSwitch*/*UAS-Stinger*. (**B**) At 1-day post-eclosion, an increase in *Stinger*-positive cells could be observed when *Stinger* only ("Stinger") was driven by *HmlΔ-GeneSwitch* (ANOVA p-value = 0.004), but not when *Stinger* + *bax* ("bax") was driven by the same driver (ANOVA p-value = 0.217). These data show that bax ablates haemocytes and the same phenotype was observed at day 1 with a constitutive driver (Fig. 1) and at 6-days post-eclosion with *HmlΔ-GeneSwitch* (Fig. 2). N = 3 flies per condition. In (**B**), and all subsequent box plots in the supplementary material, boxes represent the 25-75% range, lines represent the median, and whiskers represent the minimum-maximum range excluding outliers; outliers were not excluded in statistical analyses however.

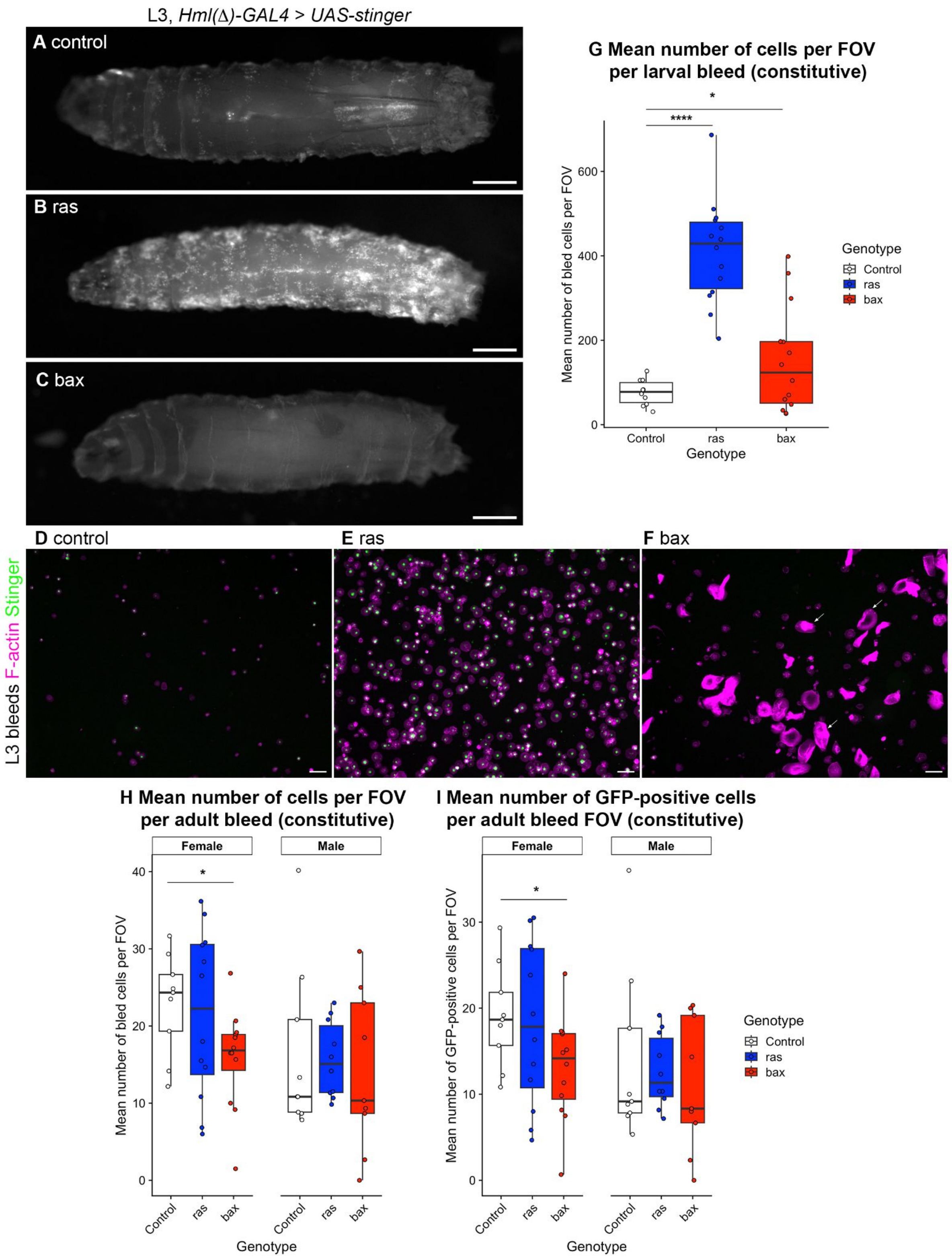

Fig. S2. Cell counts and representative images of cells bled from larvae and adults in which Stinger and either *ras85D^{V12}* or *bax* were driven by *HmlΔ-Gal4.* (**A-C**) L3 larvae with *HmlΔ-GAL4* used to drive constitutive expression of Stinger alone (control, **A**), Stinger and constitutively-active Ras85D^{V12} (ras, **B**), and Stinger and Bax (bax, **C**). GFP channel images show expression from *UAS-Stinger*; scale bars represent 500 μm; anterior is left. (**D-F**) Representative fields of view (FOV) of haemocytes dissected from control (**D**), ras (**E**) and bax (**F**) L3 larvae. Merged images show F-actin (magenta) and Stinger (green) fluorescence; arrows indicate lamellocyte-like cells in bax bleeds (**F**); scale bars represent 50 μm. (**G**) scatterplot

Journal of Experimental Biology • Supplementary information

showing average number of cells per FOV for L3 larval bleeds. (**H-I**) scatterplots showing average number of cells (**H**) and average number of GFP-positive cells (i.e., marked via *HmlΔ-GAL4*, **I**), per FOV, per adult dissection; n.b., two flies dissected per well. For all bleed data, each point represents the total cells bled from 1 fly or 1 larva. Statistical significance was calculated using Student's two-tailed *t*-tests; * and **** represent p-values ≤ 0.05 and 0.0001, respectively. All genotypes are as follows: *w^{1118};;HmlΔ-GAL4/UAS-Stinger* (control), *w^{1118};+/UAS-ras85D^{V12};HmlΔ-GAL4/UAS-Stinger* (ras), *w^{1118};+/UAS-bax;HmlΔ-GAL4/UAS-Stinger* (bax).

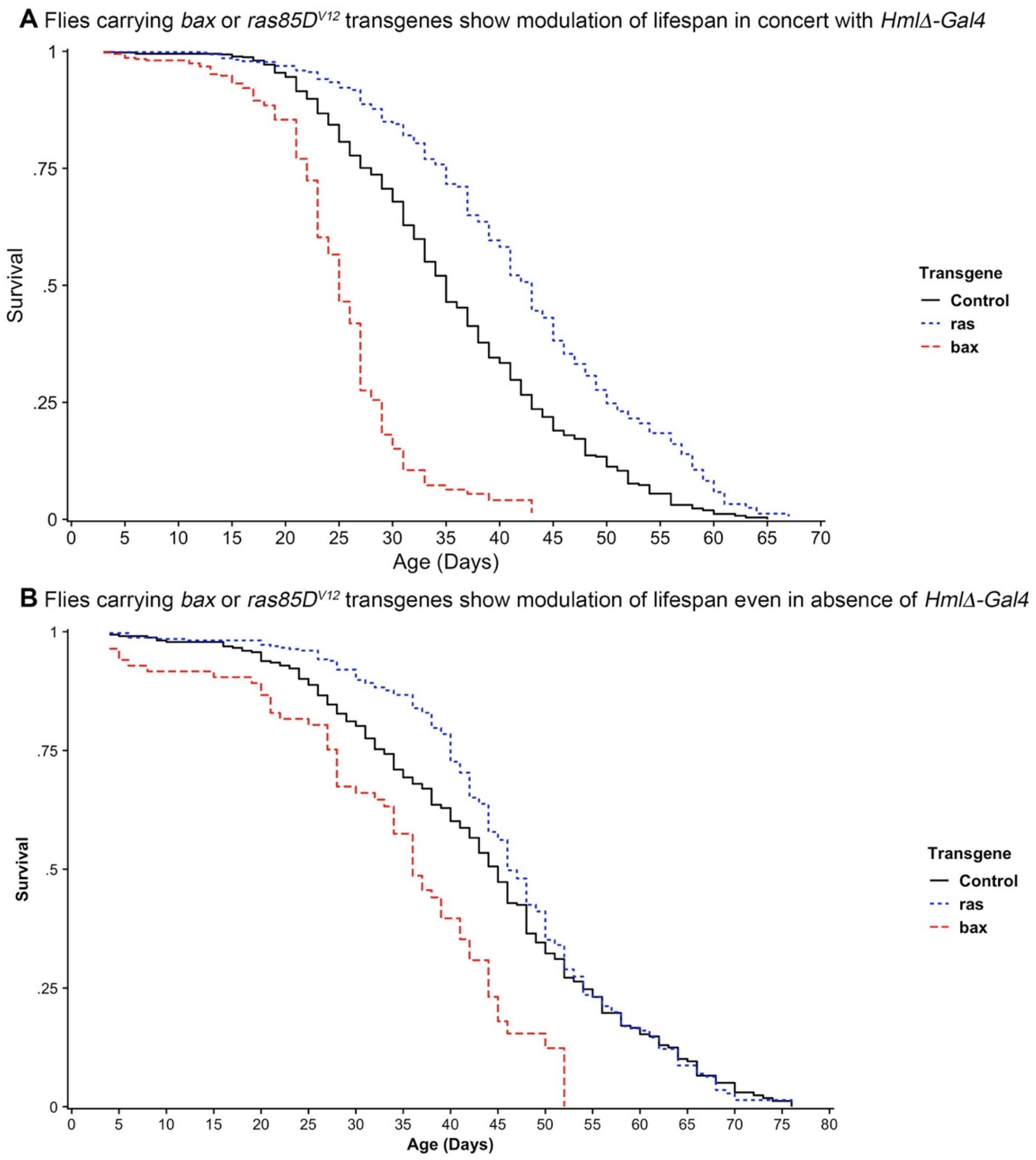

Fig. S3. Modulation of lifespan for flies carrying *bax* or *ras85D*^{V12} transgenes in the presence or absence of *HmlΔ-Gal4*. Following eclosion, flies of the indicated genotypes were mated for 48 hours. Subsequently, flies were sorted into lifespan cages, for which new food was provided every 48 hours and the number of dead flies was counted; statistical analysis was via proportional cox hazard models. (**A**) For 'Control' flies, *HmlΔ-Gal4* drove only the *UAS-Stinger* transgene, whereas for 'bax' and 'ras' flies, either *UAS-bax* or *UAS-ras85D*^{V12} were driven in addition to *UAS-Stinger*. N ≥ 360 flies for each condition, each transgene was highly statistically significant relative to the control (p-values < 1×10^{-20}). The exact genotypes were as follows: 'Control' = w^{1118};+;*UAS-Stinger/HmlΔ-Gal4*; 'ras' = w^{1118};+/*UAS-ras85D*^{V12};*UAS-Stinger/HmlΔ-Gal4*; 'bax' = w^{1118};+/*UAS-bax*;*UAS-Stinger/HmlΔ-Gal4*. (**B**) Male *UAS-bax*, *UAS-ras85D*^{V12} or control lines were crossed to *yw* females (i.e., no *HmlΔ-Gal4* was present) and lifespan assessed for their progeny. N ≥ 80 flies for each condition. P-value (bax) = 0.00002, p-value (ras) = 0.37. The genotypes used here to assess lifespan were as follows: 'Control': w^{1118}/w^{yw};+;*UAS-Stinger*/+; 'ras': w^{1118}/w^{yw};*UAS-ras85D*^{V12}/+;*UAS-Stinger*/+; 'bax': w^{1118}/w^{yw};*UAS-bax*/+;*UAS-Stinger*/+.

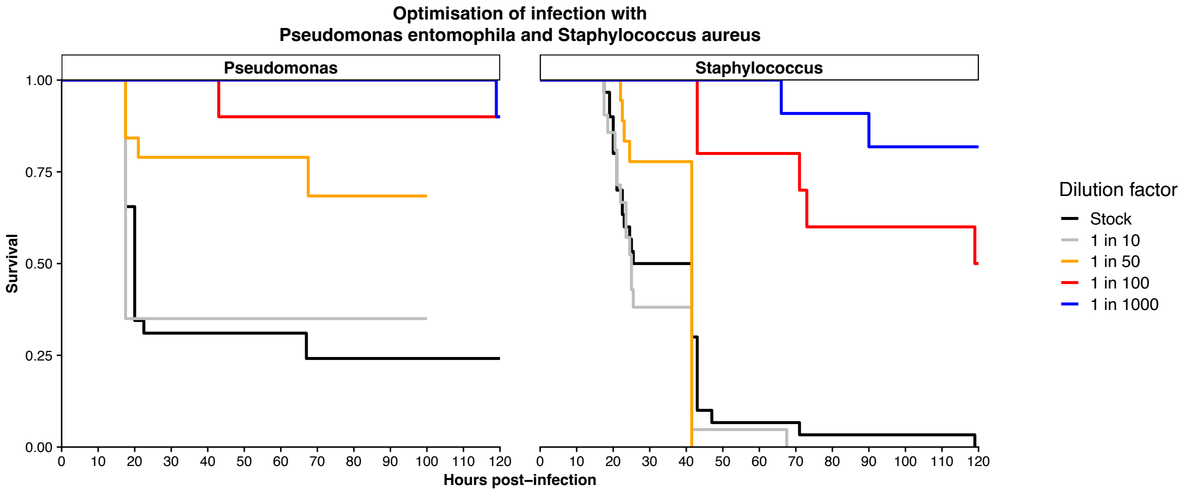

Fig. S4. Infection optimisation experiment with *Pseudomonas entomophila* and *Staphylococcus aureus* in the *HmlΔ-GeneSwitch* genotype. Flies of the genotype *w*[1118]*; +;HmlΔ-GeneSwitch* were infected with either stock or a dilution factor of either *Pseudomonas entomophila* and *Staphylococcus aureus*. The stock concentrations were individually grown overnight at 29°C in LB broth, from stocks frozen at -80°C, to an OD_{600nm} of 0.5, prior to dilution. Considering that our planned experiments required a high number of deaths in order to assess statistical significance effectively, we opted to use the stock concentrations for both bacteria in the full experiment. Experiment performed as a single replicate for the purposes of dose optimisation; all groups are ≥ 10 individuals.

Journal of Experimental Biology • Supplementary information

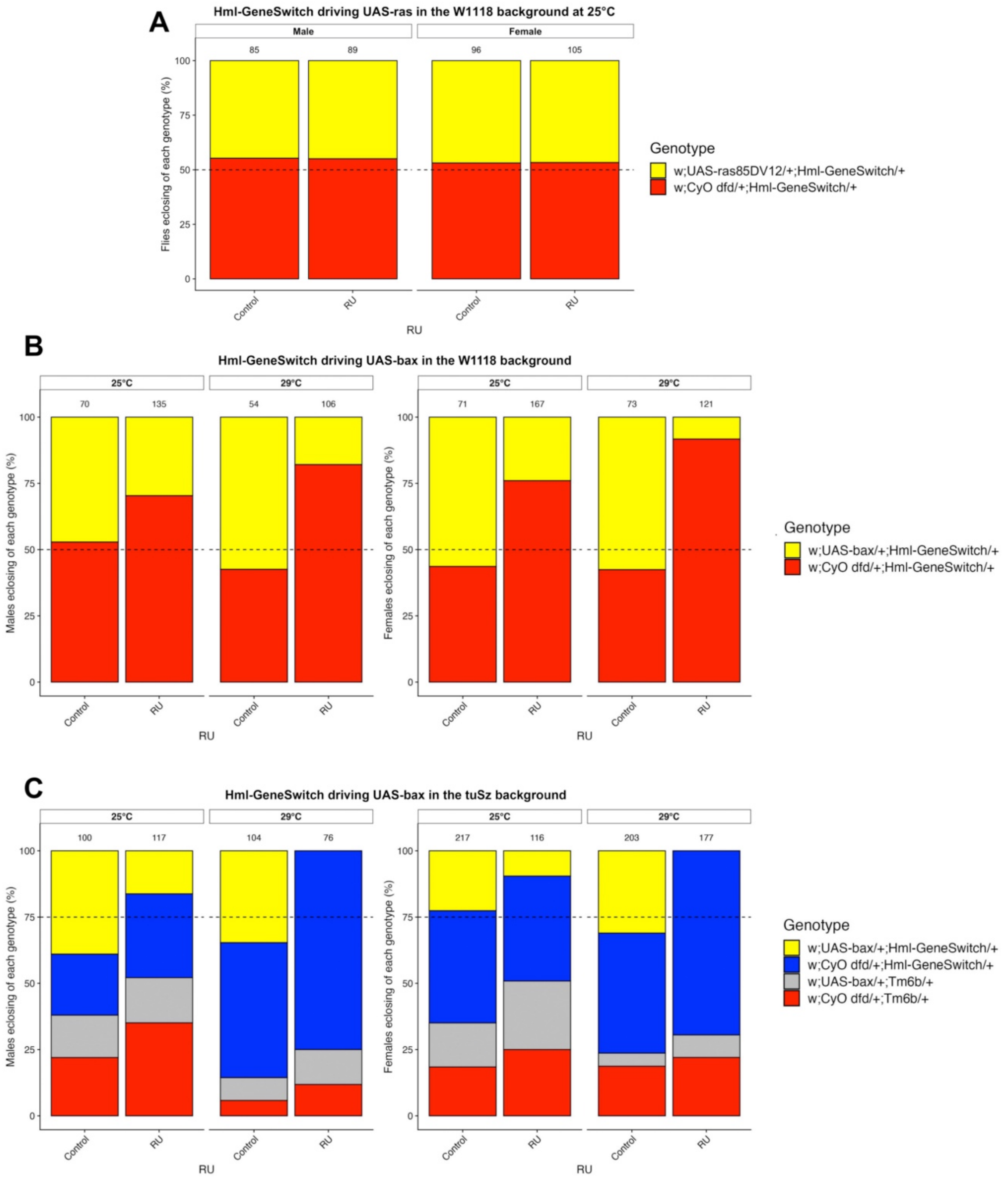

Fig. S5. Reduction of *Hml*-positive cell numbers using *UAS-bax* also reduces eclosion rates, and the magnitude of this effect is increased by both the *tuSz*^{1} genetic background and increased temperature. (**A**) Eclosion ratios in a control genetic background: female *w*^{1118}*;+;HmlΔ-GeneSwitch* flies were crossed with male *w*^{1118}*;UAS-ras85D*^{V12}*/CyO dfd* at 25°C. The number of flies eclosing with *CyO dfd* was compared to those eclosing without it in either control or RU conditions, so as to genotype the eclosing flies. The estimated ratio for each condition was 50%, indicated by the dotted line. (**B**) Female *w*^{1118}*;+;HmlΔ-GeneSwitch* flies were crossed with male *w*^{1118}*/Y;UAS-bax*/CyO *dfd* at 25°C or 29°C. The number of flies eclosing with *CyO dfd* was compared to those eclosing without it in either control or RU conditions, so as to genotype the eclosing flies. The estimated ratio for each condition was 50%, indicated by the dotted line. In this genetic background, RU provision reduced eclosion rates of flies carrying both the *HmlΔ-GeneSwitch* and *UAS-bax* transgenes. This effect was observed at both in males and females and at both 25°C and 29°C, but the effect magnitude was increased by the higher temperature. (**C**) Female *w*^{tuSz1}*;+;HmlΔ-GeneSwitch* flies were crossed with male *w*^{1118}*/Y;UAS-bax*/CyO *dfd* at

Journal of Experimental Biology • Supplementary information

25°C or 29°C. The number of flies eclosing without *CyO dfd* or *TM6b* was compared to those eclosing with these balancer chromosomes. The estimated ratio for each condition was 25%, indicated by the dotted line. RU provision reduced the eclosion rate of flies carrying both the *HmlΔ-GeneSwitch* and *UAS-bax* transgenes in all conditions, and in fact at 29°C RU conditions, no male nor female eclosers of this genotype were observed. For all panels (**A**-**C**), the total number of progeny counted from each cross is shown above each bar.

Table S1. Statistics for the effect of *Hml*-positive cell ablation and expansion on lifespan and post-infection survival using *HmlΔ-GeneSwitch*. (**A**) Statistics for effects on lifespan, calculated using the Coxme R package. (**B**) Statistics for effect on post-infection survival, calculated using the Coxph R package. For (**A-B**) each sample is a single fly; HR is the hazard ratio.

A) Statistics for effect of *Hml* -positive cell ablation and expansion on lifespan using *HmlΔ-GS*

		RU vs control statistics			Sample sizes	
**Condition**	**Sex**	**ln(HR)**	**se(HR)**	**P-value**	**Control**	**RU**
Control	Female	0.865	0.081	0.075	827	554
bax	Female	0.949	0.094	0.577	282	253
ras	Female	1.074	0.081	0.381	404	335
Control	Male	1.11	0.06	0.083	685	729
bax	Male	0.943	0.088	0.509	270	313
ras	Male	0.865	0.081	0.074	401	335

B) Statistics for effect of *Hml* -positive cell ablation and expansion on post-infection survival using *HmlΔ-GS*

		RU vs control statistics			Sample sizes	
**Condition**	**Bacteria**	**ln(HR)**	**se(HR)**	**P-value**	**Control**	**RU**
Control	*P. entomophila*	1.231	0.361	0.565	23	15
bax	*P. entomophila*	0.574	0.379	0.143	16	18
ras	*P. entomophila*	1.037	0.359	0.92	22	23
Control	*S. aureus*	0.675	0.335	0.241	21	18
bax	*S. aureus*	0.46	0.402	0.053	14	18
ras	*S. aureus*	0.658	0.326	0.198	20	22
